# Berberine Alleviates Non-alcoholic Steatohepatitis Through Modulating Gut Microbiota Mediated Intestinal FXR Activation

**DOI:** 10.3389/fphar.2021.750826

**Published:** 2021-09-17

**Authors:** Xiangbing Shu, Meng Li, Ying Cao, Chunlin Li, Wenjun Zhou, Guang Ji, Li Zhang

**Affiliations:** ^1^ Institute of Digestive Diseases, Longhua Hospital, Shanghai University of Traditional Chinese Medicine, Shanghai, China; ^2^ Department of Geratology, Baoshan Branch of Shuguang Hospital, Shanghai University of Traditional Chinese Medicine, Shanghai, China

**Keywords:** berberine, non-alcoholic steatohepatitis (NASH), gut microbiota, bile acids, farnesoid X receptor (FXR)

## Abstract

Berberine is a natural plant alkaloid isolated from a diverse range of genera, it obtains anti-inflammatory, anti-obesity, and hepatoprotective properties, and is a promising agent for non-alcoholic steatohepatitis (NASH). Farnesoid X receptor (FXR) is a bile acid receptor and a drug target for NASH, however, the underlying mechanisms of berberine on regulating FXR are still unknown. In the present study, we feed mice with a 12-week high-fat diet with interval dextran sulfate sodium (0.5% in drinking water) diet to induce NASH, and treat the mice with berberine (100 mg/kg per day) *via* oral gavage for additional 4 weeks. We demonstrate that administration of berberine alleviates steatosis and infiltration of inflammatory cells in the liver of NASH mice. We apply 16S ribosomal DNA sequencing to screen the structure of gut microbiota, and ultra-performance liquid chromatography-tandem mass spectrometry analysis to determine the bile acid profiles. The results show that berberine modulates gut dysbiosis, and specifically increases the relative abundance of *Clostridiales*, *Lactobacillaceae,* and *Bacteroidale*. Berberine modulated microbiomes are associated with bile acid de-conjugation and transformation, which are consistent with the altered bile acid species (e.g., deoxycholic acid, ursodeoxycholic acid) upon berberine treatment. BA species that respond to berberine treatment are known FXR agonists, thus we performed quantitative Real Time-PCR and western blot to examine the FXR pathway, and find that berberine up-regulates intestinal FXR and fibroblast growth factor 15 (FGF15) expression, and the secretion of FGF15 further inhibits lipogenesis and nuclear factor-κB activation in the liver. Whereas the beneficial effects of berberine are blunted in FXR knockout mice. Our results reveal that berberine alleviates NASH by modulating the interplay of gut microbiota and bile acid metabolism, as well as the subsequent intestinal FXR activation.

## Introduction

Nonalcoholic fatty liver disease (NAFLD) is becoming a world epidemic currently, the histopathologic spectrum of NAFLD includes simple steatosis, nonalcoholic steatohepatitis (NASH), fibrosis, and hepatocellular carcinoma (Pataky et al., 2016). Although simple steatosis is considered to be benign, the presence of inflammation and injury in NASH usually indicates the liver injury (Takaki et al., 2014), and approximately 30% of NASH patients could eventually develop into cirrhosis or hepatocellular carcinoma (Adams et al., 2005; Ekstedt et al., 2006). Dietary management and lifestyle modification are the first-line recommendations for NASH treatment, however, many patients are difficult to maintain the pattern for a prolonged period of time, making pharmaceutical therapy in urgent need. Since the mechanisms of NASH are profound and are only partially understood, there are no approved drugs in the NASH field.

Berberine (BBR) is a natural product that is principally isolated from herbs such as *Coptis chinensis* Franch. [Ranunculaceae], *Phellodendron chinense* C.K.Schneid. [Rutaceae], *Hydrastis canadensis* L. [Ranunculaceae], *Berberis aquifolium* Pursh [Berberidaceae], and *Berberis vulgaris* L. [Berberidaceae] (Neag et al., 2018). BBR is initially used as an anti-microbial agent for infective diarrhea. Since the 1980s, BBR has been reported to be beneficial for metabolic disorders such as type 2 diabetes (Yin et al., 2008; Lan et al., 2015; Belwal et al., 2020), dyslipidemia (Guo et al., 2019), NAFLD/NASH (Zhou et al., 2019) among others. However, the effects of BBR contrast with its low bioavailability, thus gut microbiota, as well as microbiota-derived metabolites, are proposed as the critical mechanisms for BBR (Zhang et al., 2020). The reciprocal interaction of bile acids (BAs) and gut microbiota has long been recognized. BAs undergo enterohepatic circulation several times per day, the primary BAs that are produced in the liver can convert to secondary BAs by the action of microbiomes, whereas the anti-microbial properties of BAs determine the growth of certain microbial species. BA receptor farnesoid X receptor (FXR) is a promising drug target for NASH, and FXR agonists are currently tested in clinical trials for NASH (Gonzalez et al., 2016; Schmidt et al., 2017). While the unwanted side effects of exogenous FXR agonists are difficult to avoid, endogenous FXR activation might be an alternative choice. Studies proposed that BBR administration decreased the enrichment of *Clostridium*, and inhibited the deconjugation of primary BA, e.g., taurine conjugated cholic acid (TCA), which activated intestinal FXR in physiologically healthy and obese mice (Sun et al., 2017; Tian et al., 2019). However, whether the regulation of BBR on gut microbiota and BA metabolism activates intestinal FXR in NASH is still unknown.

In the present study, we show that BBR administration potently improves hepatic steatosis and reduces infiltration of inflammatory cells in the liver of NASH mice. Applying 16S ribosomal DNA (16SrDNA)-based microbiota analysis and BA profiling, we demonstrate that BBR treatment remodels the structure of gut microbiota and alters the composition of BA species in NASH mice. The increase of certain BA species, e.g., deoxycholic acid (DCA), chenodeoxycholic acid (CDCA) in the intestine might activate FXR-fibroblast growth factor 15 (FGF15) axis, and further suppresses lipogenesis and inflammation in the liver. We also demonstrate that the beneficial effects of BBR are abolished in FXR knock-out mice. These findings thus suggest that intestinal FXR activation is one of the underlying mechanisms of BBR on NASH mice.

## Materials and Methods

### Chemicals and Reagents

Berberine hydrochloride was purchased from Shanghai Source Leaf Biological Technology Co., Ltd. (Shanghai, China), the purity of BBR is ≥98%. Oil red O reagent, high-performance liquid chromatography grade formic acid and ammonium acetate, and BA standards including cholic acid (CA), CDCA, DCA, ursodeoxycholic acid (UDCA), α, β and ω muricholic acids (MCA), tauroursodeoxycholic acid (TUDCA), taurochenodeoxycholic acid (TCDCA), tauro α-muricholic acid (TαMCA), tauro β-muricholic acid (TβMCA), tauro ω-muricholic acid (TωMCA), TCA, and taurodeoxycholic acid (TDCA) were obtained from Sigma-Aldrich (St. Louis, Mo, United States) and the purity of the above reagents was higher than 98%.

### Animals

Male C57BL/6J mice (6–8 weeks old, 22 ± 2 g) were purchased from SLAC Laboratory Animal Co., Ltd. (Shanghai, China). Male FXR knockout (FXR^−/-^) mice on a C57BL/6J background were given as a present by Prof. Li Yang from Shanghai University of Traditional Chinese medicine. After 1-week acclimatization, the mice were fed with chow diet (normal control group, *n* = 8) or high-fat diet (HFD, 60% of calories derived from fat, Research Diets, NJ, United States) supplemented with 1% dextran sulfate sodium (DSS) in drinking water periodically (7-day DSS administration followed by a 10-day interval). After 12 weeks of the diet, the HFD-DSS fed C57BL/6J mice were then randomly divided into NASH group (vehicle control, continuously supply of HFD-DSS, *n* = 8) and BBR-treated group (with HFD-DSS and BBR treatment, *n* = 8). The LD50 of BBR is 713.58 mg/kg in mice, and the present study applied the routine dosage of 100 mg/kg/d to the NASH mice. BBR was suspended in 0.5% carboxymethyl cellulose sodium solution (CMC-Na) and administered to the mice by gavage (0.1 ml/10 g body weight) once a day for 4 weeks, the normal control and vehicle control (NASH) mice were given an equal volume of 0.5% CMC-Na solution. For HFD-DSS fed FXR^−/−^mice, BBR and vehicle treatment were the same as C57BL/6J mice. All animals were maintained under controlled temperature (25°C ± 2°C) and humidity (60 ± 5%) at a 12 h light-dark cycle, and access to food and water *ad libitum*. Food consumption and body weight were recorded weekly. The animal experiments were all approved by the Animal Experiment Ethics Committee of Shanghai University of Traditional Chinese Medicine.

### Serum and Liver Biochemical Parameters Analysis

At the end of the experiment, all animals were fasted for 12 h, anesthetized intraperitoneally with 2% pentobarbital sodium, and sacrificed. To obtain the serum samples, blood was collected and centrifugated at 4°C, 3,000 rpm for 15 min. Serum lipid profiles including triglyceride (TG), total cholesterol (TC), low-density lipoprotein cholesterol (LDL-c), and serum enzymes as indicators of liver damage such as alanine transaminase (ALT) and aspartate transaminase (AST) were analyzed using the Hitachi full-automatic system.

For measurement of hepatic TG content, liver tissues were rapidly excised and rinsed with precooled normal saline, the same portion of liver samples were cut and homogenized with 10 volumes of ice-cold ethanol in a homogenizer. The homogenate was extracted overnight at 4°C and centrifuged at 4°C, 3,000 rpm for 15 min. The organic layer was removed and the content of TG and TC were measured using commercial kits from Nanjing Jiancheng Bioengineering Institute (Nanjing, China) according to the manufacturer’s instruction.

### Liver Histopathologic Evaluation

Liver histological changes were examined according to the previously described method (Li Q. et al., 2020). Briefly, for hematoxylin and eosin (H&E) staining, liver tissues were fixed in 10% formalin solution for 24 h, dehydrated, paraffin-embedded, sectioned to ∼5 μm thickness, and stained with H&E reagent (Kohypath, Shanghai, China). For Oil Red O (ORO) staining, frozen liver tissues were embedded in Tissue-Tek OCT Compound (Sakura, Tokyo, Japan), cut into ∼8 μm frozen sections, and stained with ORO reagent (Sigma, St. Louis, MO, United States). Images were captured under a Nikon Eclipse 50i microscope (Nikon, Tokyo, Japan) with a magnification of 200×.

### Fecal 16S rDNA Analysis

Fecal samples were collected from the cecum of the mice, and total DNA in feces was isolated using the Qiagen QIAmp® Fast DNA Stool Mini Kit (Qiagen, CA, United States) according to the recommended protocol. The extracted DNA from each sample was used as a template to amplify the V3-V4 region of bacterial 16S rDNA genes of distinct regions was carried out by PCR, and then further gel-purified and quantified. Purified amplicons were pooled in equimolar and sequenced on the Illumina MiSeq^TM^ platform (paired-end) by Majorbio Bio-Pharm Technology Co. Ltd. (Shanghai, China). The raw sequencing reads were generated and quality-filtered by QIIME version 1.9.1 with optimized parameters and analyzed using Mothur version 1.33.0 (http://www.mothur.org/), UPARSE version 7.1 (http://drive5.com/uparse/) and R version 3.6.1 (http://www.R-project.org/). The raw data of 16S rDNA was deposited at https://www.ncbi.nlm.nih.gov/Traces/study/?acc=PRJNA753953.

### Bile Acids Profiling Analysis Using Ultra-Performance Liquid Chromatography Tandem-Mass Spectrometry

Serum, liver, and fecal samples were collected, and the BAs were extracted and quantified by ultra-performance liquid chromatography tandem-mass spectrometry (UPLC/MS) according to our previous research (Li Q. et al., 2020).

### Western Blot Analysis

Liver tissues were lysed with radioimmunoprecipitation assay lysis buffer (Beyotime Institute of Biotechnology, Shanghai, China) with protease and phosphatase inhibitors (Roche, Indiana, United States). The prepared protein was separated by 12% SDS-PAGE, transferred to polyvinylidene difluoride membranes (Millipore, Temecula, CA, United States), and incubated with the primary antibody and secondary antibody, respectively. Anti-FXR, anti-FGF15, and anti-small heterodimer partner (SHP) were obtained from Abcam (Cambridge, United Kingdom), anti-cholesterol 7-alpha hydroxy-lase (CYP7A1) and anti-cytochrome P450, family 8, subfamily B, polypeptide 1 (CYP8B1) were purchased from Santa Cruz Biotechnology (Texas, CA, United States), anti-nuclear factor-κB (NF-κB) p65 and anti-NF-κB P-p65 were purchase from Cell Signaling Technology (Beverly, MA, United States). As an internal control, β-actin was purchased from HuaBio (Hangzhou, China). Anti-rabbit IgG and anti-mouse IgG was obtained from Cell Signaling Technology (Beverly, MA, United States). The bands were visualized by ECL chemiluminescence detection kit (Millipore, MA, United States), and quantified using the Tanon 5200 Chemiluminescent Imaging System (Tanon Science & Technology Inc., Shanghai, China).

### Quantitative Real Time-PCR Analysis

Quantitative Real Time-PCR (RT-qPCR) was conducted as described previously (Li Q. et al., 2020). Briefly, total RNA was extracted from liver and intestinal tissues using TRIzol reagent (Thermo Fisher Scientific, United States), reversely transcribed into cDNA by reverse transcription kits (Promega, Madison, WI, United States), and subjected to PCR on a StepOne Applied PCR system (Applied Biosystems, Carlsbad, CA, United States) using PowerUp SYBR Green Master Mix (TOYOBO, Osaka, Japan). The relative gene expression was normalized to glyceraldehyde-3-phosphate dehydrogenase (GAPDH) using a 2^−ΔΔCt^ method. The gene function and primer sequences were listed in Table 1.

**TABLE 1:** Primers used for mRNA analysis.

Genes	Gene function	Forward primer	Reverse primer
GAPDH	Internal control	GTGCCGCCTGGAGAAACC	GGT​GGA​AGA​GTG​GGA​GTT​GC
FXR	BA receptor	CGG​CTG​TCA​GGA​TTT​GTG​C	GAA​GCC​CAG​GTT​GGA​ATA​GTA​AG
SHP	Form heterodimer with FXR	AACCTGCCGTCCTTCTGC	GAGCCTCAGCCACCTCGA
FGF15	Feedback inhibition of BA synthesis	GAT​CCA​CTC​TTT​CTC​TAC​GGC​TG	CGT​TCG​TTT​TGG​TCC​TCC​TC
FGFR4	FGF15 receptor	CTG​TAT​GGG​CTA​ATG​AGG​GAG​TG	TCA​GGC​GGA​GGT​CAA​GGT​AC
CYP7A1	BA synthesis enzyme	TTC​AAG​ACC​GCA​CAT​AAA​GCC	GAG​ATG​CCC​AGA​GGA​TCA​CG
CYP8B1	BA synthesis enzyme	CTC​GGG​TGT​TTC​CAA​GTG​C	GGG​CTT​CAG​GCG​ATA​GAG​G
SREBP1	Transcription of lipogenesis	AGT​CCA​GCC​TTT​GAG​GAT​AGC​C	CCG​TAG​CAT​CAG​AGG​GAG​TGA​G
ACC	Key enzyme in fatty acid synthesis	CCA​CAG​AAC​TTA​CAA​GGC​ACG	GAATTGTGAGGGTCGGCG
FASN	Key enzyme in fatty acid synthesis	GTC​AAC​AAC​CAT​AGG​CGA​TTT​C	GCA​CCC​TGA​CCC​AGA​ATA​CC
SCD1	Key enzyme in fatty acid synthesis	GAC​CTG​AAA​GCC​GAG​AAG​C	ATG​AAG​CAC​ATC​AGC​AGG​AGG
TNF-α	Inflammatory cytokine	ACG​TGG​AAC​TGG​CAG​AAG​AG	GGT​TGT​CTT​TGA​GAT​CCA​TGC
IL-6	Inflammatory cytokine	AAA​TGA​TGG​ATG​CTA​CCA​AAC​TG	CTC​TGG​CTT​TGT​CTT​TCT​TGT​TAT​C

### Statistical Analysis

The quantitative data were presented with mean ± SEM and analyzed by one-way analysis of variance (ANOVA) and unpaired two-tailed Student’s *t*-test using SPSS 18.0 statistical software (Chicago, IL, United States) and GraphPad Prism version 8.0 (San Diego, CA, United States). *p* < 0.05 was considered statistically different.

## Results

### Berberine Alleviates Non-Alcoholic Steatohepatitis in HFD-DSS Induced Mice

**FIGURE 1:**
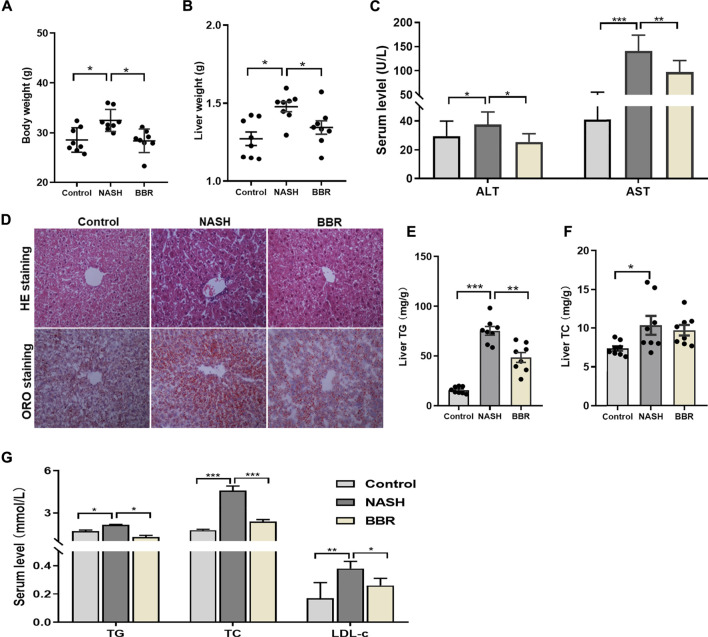
Effects of BBR on HFD-DSS induced NASH mice. **(A)** Body weight; **(B)** Liver weight; **(C)** Serum ALT and AST levels; **(D)** Representative H&E and ORO staining images of liver sections (magnification 200×); **(E,F)** Liver TG and TC content; **(G)** Serum TG, TC and LDL-c levels. Data are expressed as mean ± SEM, eight animals are allocated for each group. *p < 0.05, **p < 0.01; ***p < 0.001 between groups.

To address the effects of BBR on NASH, we used HFD-DSS induced NASH model. The treatment of DSS is shown to increase portal liposaccharide (LPS) level and therefore exacerbates the progression of NASH (Gabele et al., 2011; Achiwa et al., 2016). Mice fed with HFD-DSS demonstrated a typical NASH phenotype as evidenced by increased body weight and liver weight, obvious hepatic steatosis and inflammation, as well as increased liver TG content. During the 4-week treatment, all the mice were well-groomed, alert, active and showed good condition. The stool color of BBR-treated mice was a little bit yellow due to the drug administration. BBR treatment significantly reduced body weight and liver weight by 10 and 8%, respectively (Figures 1A,B), improved hepatic steatosis and inflammation (Figures 1C,D), and reduced liver TG content by approximately 30% (Figure 1E). The serum levels of AST and ALT in NASH mice were significantly increased, and BBR treatment could decrease the serum ALT and AST levels by 30% each (Figure 1C). Assembly, serum TG, TC, and LDL-c levels were increased in NASH mice, and BBR treatment partially restored these parameters (Figure 1F). These results implied that BBR exhibits robust efficacy against NASH in HFD-DSS fed mice.

### Berberine Reshaped Gut Microbiota in Non-Alcoholic Steatohepatitis Mice

**FIGURE 2:**
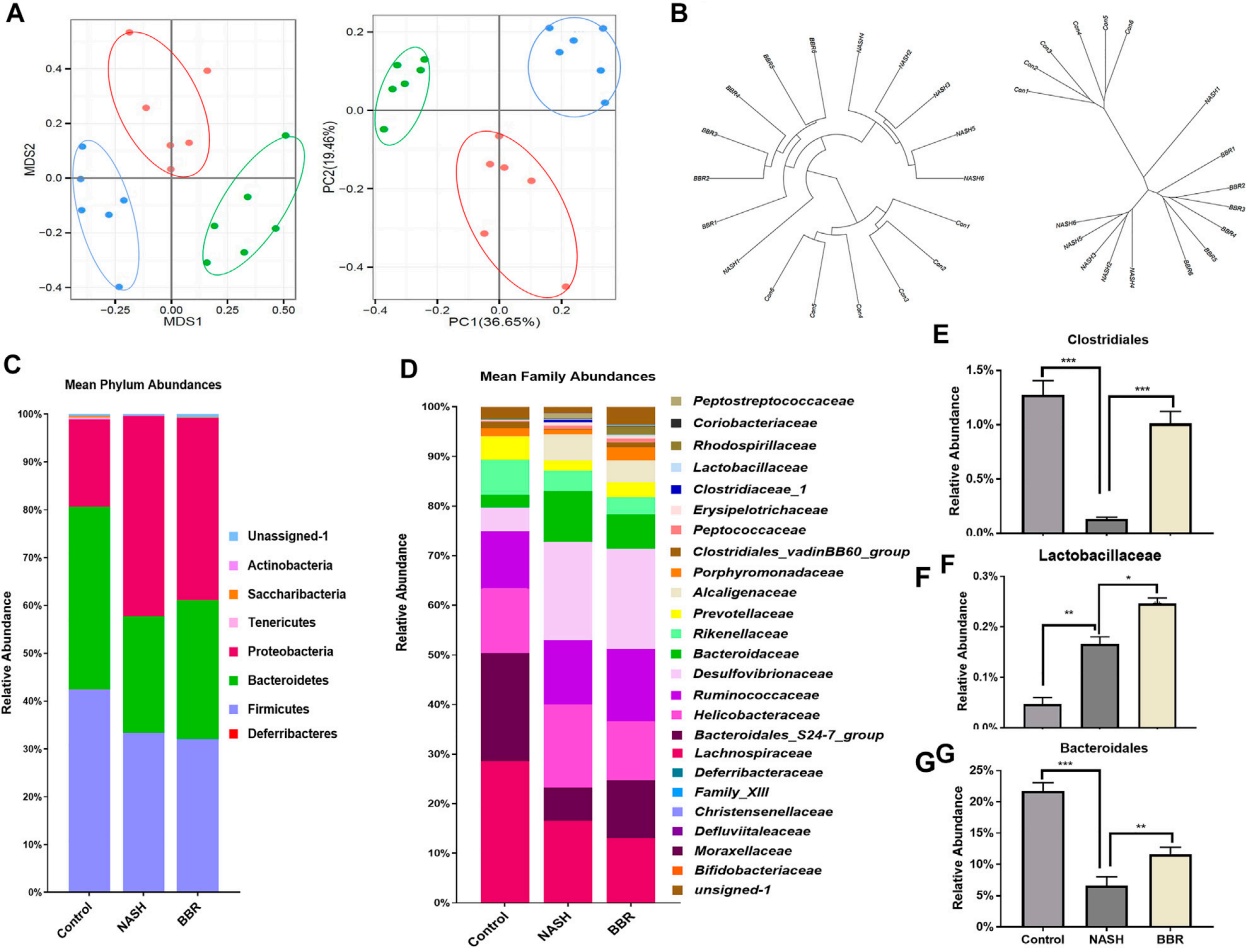
BBR reshapes gut microbiota in NASH mice. **(A)** PCoA analysis based on the unweighted UniFrac analysis of OTUs; **(B)** Bray-Curtis distance analysis of OTUs; **(C)** Mean phylum abundance of OTUs; **(D)** Mean family abundance of OTUs; **(E–G)** The relative abundance of *Clostridiales*, *Lactobacillaceae* and *Bacteroidaies*. Data are expressed as mean ± SEM of six animals per group. *p < 0.05, **p < 0.01; ***p < 0.001 between groups.

Gut dysbiosis is associated with the pathogenesis of NASH, and modulating gut microbiota is reported to contribute to the efficacy of BBR in various diseases. Here we analyzed the overall structural changes of gut microbiota in response to BBR by 16S rDNA sequencing. A total of 3,274 operational taxonomic units (OTUs) were captured. UniFrac distance-based principal coordinate analysis (PCoA) (Figure 2A) and Bray-Curtis distance (Figure 2B) analysis revealed distinct clustering of microbe communities among control mice, NASH mice, as well as BBR treated mice. To assess the overall composition of the bacterial community in different groups, we analyzed the degree of bacterial taxonomic similarity at the phylum level. Compared to control mice, NASH mice displayed a higher Firmicutes-to-Bacteroidetes ratio due to the significant decrease in the relative abundance of Bacteroidetes, whereas BBR treatment increased the relative abundance of Bacteroidetes (Figure 2C). At the family level, BBR treatment significantly increased the relative abundance of *Clostridiales*, *Lactobacillaceae*, and *Bacteroidale* (Figures 2D–G). Assembly, these increased bacteria are participating in bile salt hydrolase (BSH) activity and secondary BA production, suggesting that BBR reshapes gut microbiota and might affect BA profiles in NASH mice.

### Berberine Altered Bile Acids Profiles in Non-Alcoholic Steatohepatitis Mice

**FIGURE 3:**
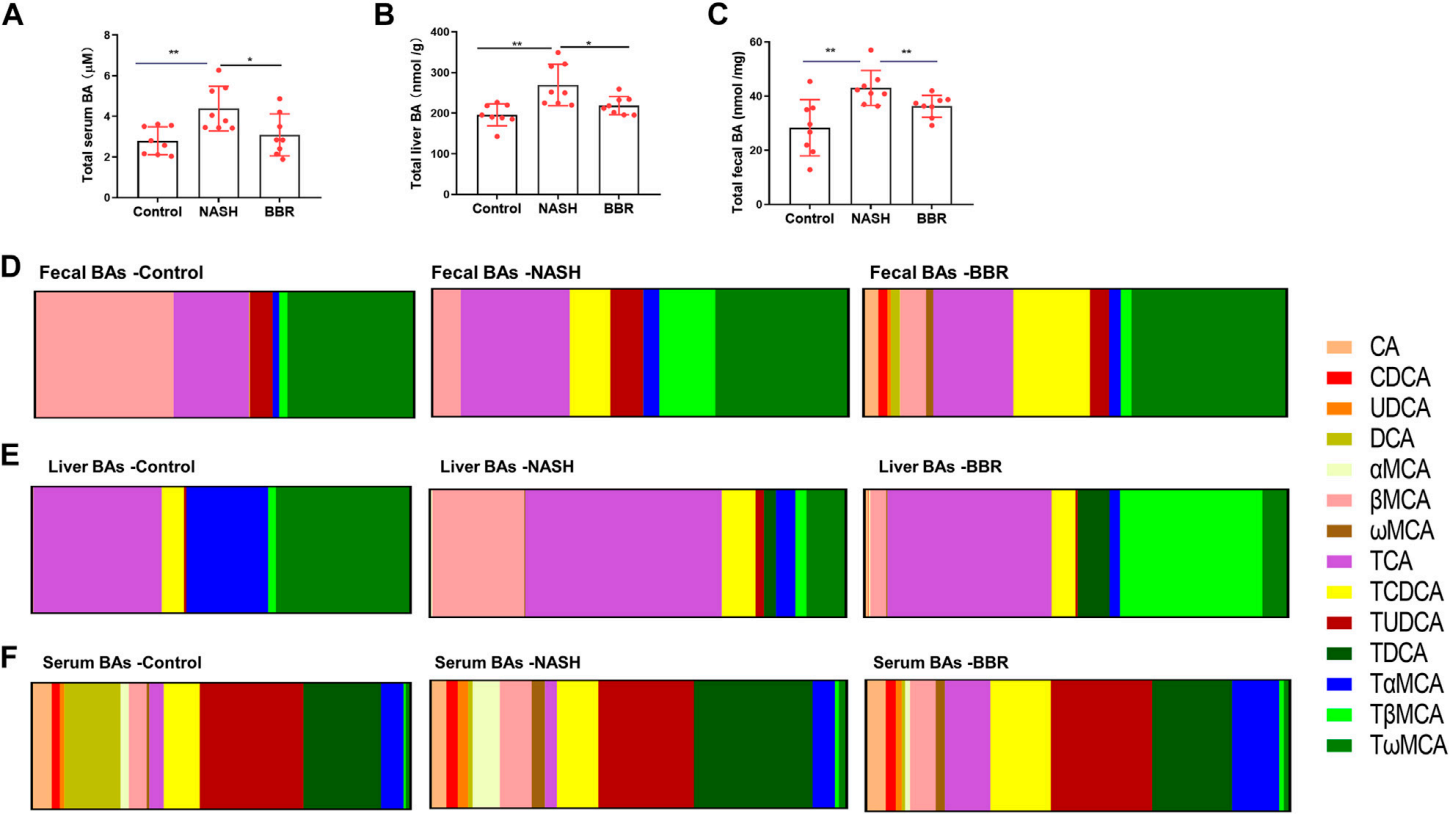
BBR alters BA profiles in NASH mice. The total BA levels in the **(A)** liver, **(B)** serum and **(C)** feces. The ratio of BA species of **(A)** feces, **(B)** liver and **(C)** serum in control, NASH and BBR-treated mice. Data are presented as mean ± SEM (*n* = 8 per group). *p < 0.05, **p < 0.01 between groups.

The interaction of gut microbiota and BA metabolism has been reported in NASH development and progression (Sydor et al., 2020). Enriched BSH producing microbiomes upon BBR treatment indicated that the BA profiles might alter accordingly. BBR treatment decreased the total BA levels in the liver (Figure 3A) and serum (Figure 3B) and increased the content of fecal BAs (Figure 3C). BA profiling (Supplementary Tables S1–S3) showed that fecal CDCA, UDCA, DCA, and MCA of NASH mice were all significantly increased in response to BBR treatment, whereas the conjugated BAs in feces were unchanged (TDCA, TCA, TωMCA) or decreased (TβMCA) upon BBR treatment (Figure 3D). BBR remarkably decreased the percentage of liver βMCA and TβMCA (Figure 3E), but had no obvious effects on serum BA profiles (Figure 3F). These results suggest that BBR promotes intestinal unconjugated and secondary BA production in NASH mice.

### Berberine Activated Intestinal Farnesoid X Receptor Signaling

**FIGURE 4:**
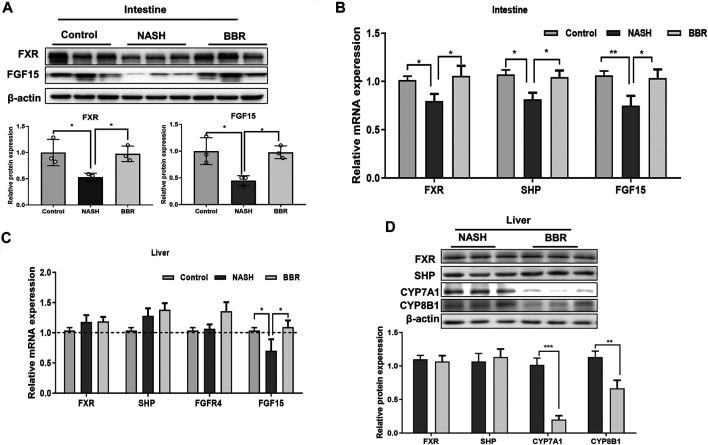
BBR regulates FXR signaling in NASH mice. **(A)** The protein expression of intestinal FXR and FGF15, and **(B)** the mRNA expression of FXR, SHP and FGF15. **(C)** Liver mRNA expression of FXR, SHP, FGFR4 and FGF15. **(D)** The protein expression of FXR, SHP, CYP7A1 and CYP8B1 between NASH mice and BBR treated mice. Data are presented as mean ± SEM (*n* = 3 per group). *p < 0.05, **p < 0.01, ***p < 0.001 between groups.

Certain BA species (e.g., DCA, UDCA) that are mediated by BBR are known FXR agonists, so we next investigated the protein expression of intestinal FXR in mice. The protein expression of intestinal FXR showed a 50% decrease in NASH mice, and BBR treatment could reverse the decrease of FXR expression (Figures 4A,B, Supplementary Figure S1). Up-regulation of intestinal FXR promotes the production of FGF15 in epithelium cells, and BBR-treatment prevented the decrease of intestinal FGF15 levels in NASH mice. Intestine-derived FGF15 may enter the liver through the portal vein, and the liver FGF15 expression was also significantly increased upon BBR treatment. We also detected the mRNA expression of liver FXR, SHP, and FGFR4, but did not find any statistical difference between NASH mice and BBR treated mice (Figure 4C). FGF15 is a suppressor of BA synthesis, and CYP7A1 and CYP8B1 are the main enzymes of BA synthesis that could convert cholesterol into CA, CDCA, and β-MCA. We found that the protein expression of CYP7A1 and CYP8B1 of BBR treated mice were all significantly decreased in comparison to untreated NASH mice, whereas the expression of liver FXR and SHP was not statistically different between BBR treated mice and untreated NASH mice (Figure 4D; Supplementary Figure S2). These results indicate that the activation of intestinal but not liver FXR inhibits the BA synthesis.

### Berberine Suppressed Liver Inflammation

**FIGURE 5:**
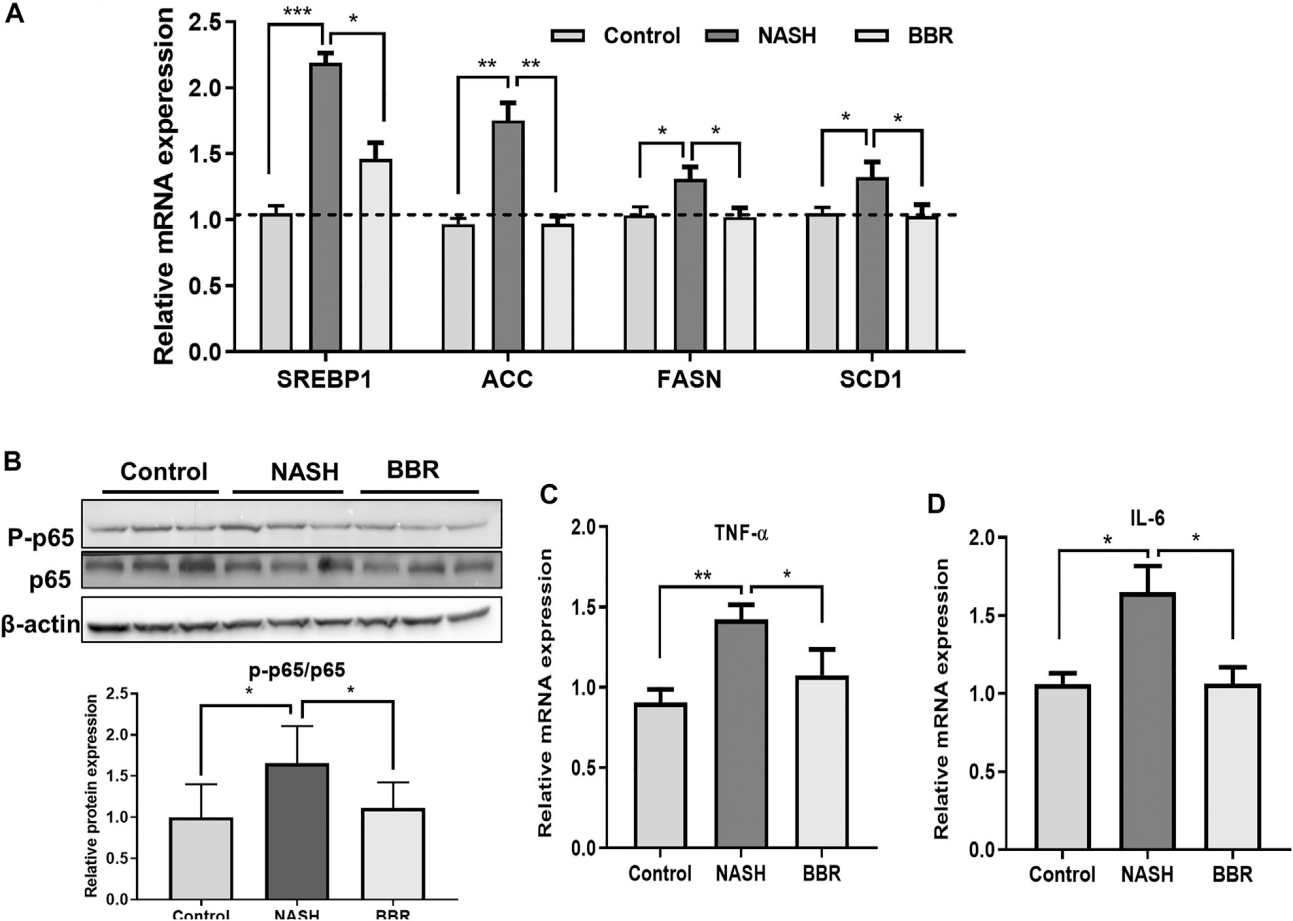
BBR suppresses liver inflammation in NASH mice. **(A)** The mRNA expression of SREBP1c, ACC, FASN and SCD1 in the liver; **(B)** The protein expression of p65 and P-p65 in the liver; **(C,D)** The mRNA expression of TNF-α and IL-6 in the liver. Data are presented as mean ± SEM (*n* = 3 per group). *p < 0.05, **p < 0.01; ***p < 0.001 between groups.

To confirm the effect of BBR on intestinal FXR activation and FGF15 secretion, we detected the downstream lipogenesis genes in the liver, and found that BBR treatment significantly decreased the mRNA expression of sterol-regulatory element-binding protein 1 (SREBP1), acetyl-CoA carboxylase (ACC), fatty acid synthase (FASN), and stearoyl-CoA desaturase-1 (SCD1) in NASH mice (Figure 5A), suggesting the suppression of *de novo* lipogenesis upon BBR treatment. In addition, BBR treatment decreased the ratio of phosphorylated p65 to total p65 (Figure 5B; Supplementary Figure S3), indicating the suppression of NF-κB activation in the liver. Accordingly, the mRNA expression of inflammatory cytokines tumor necrosis factor alpha (TNF-α) (Figure 5C) and interleukin-6 (IL-6) (Figure 5D) was also decreased in BBR treated mice. These results indicate that BBR suppresses lipogenesis and inflammatory pathways *via* intestinal FXR activation.

### The Effect of Berberine was Abolished in FXR^−/−^ Mice

**FIGURE 6:**
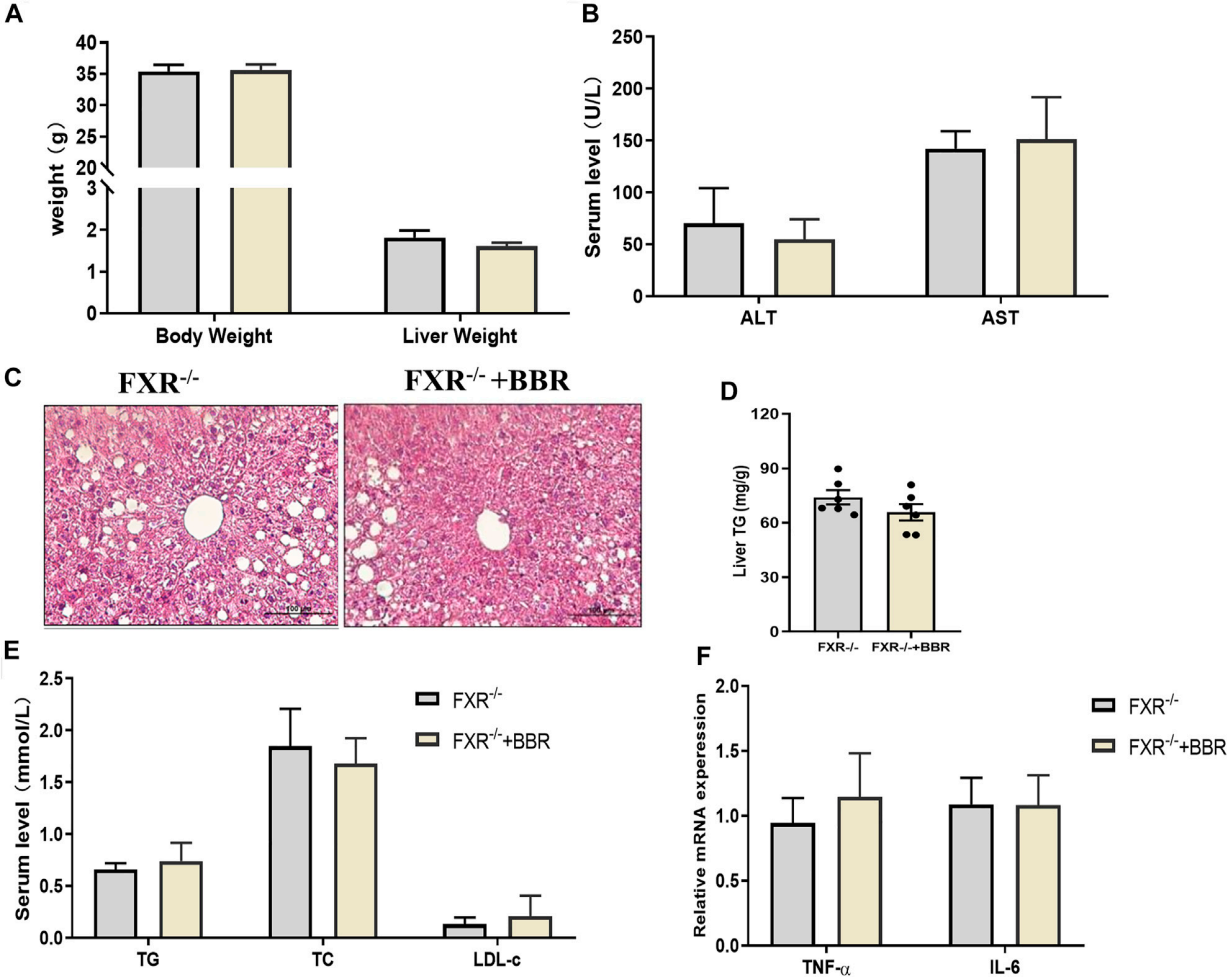
The effect of BBR on FXR^−/−^ mice. **(A)** The body weight and liver weight. **(B)** Serum ALT and AST levels. **(C)** Representative H&E staining images of liver sections (magnification 200×). **(D)** Liver TG content. **(E)** Serum TG, TC and LDL-c levels. **(F)** The mRNA expression of TNF-α and IL-6 in the liver. Data are presented as mean ± SEM (*n* = 6 per group).

To confirm the effect of BBR on FXR, we conducted experiments on FXR^−/−^ mice. The results showed that BBR treatment has no effect on body weight and liver weight in FXR^−/−^ mice (Figure 6A). The serum levels of ALT and AST were also comparable between BBR treated and untreated FXR^−/−^ mice (Figure 6B). FXR^−/-^ mice demonstrated obvious hepatic steatosis and inflammatory cell infiltration in the liver, whereas the effects of BBR on improving hepatic steatosis were negligible in FXR^−/−^ mice (Figures 6C,D). Consistently, the serum levels of TG, TC, and LDL-c, as well as the mRNA expressions of TNF-α and IL-6 were not statistically different between BBR treated and untreated FXR^−/−^ mice (Figures 6E,F). These results suggest that the beneficial effects of BBR are abolished in FXR^−/-^ mice.

## Discussion

**FIGURE 7:**
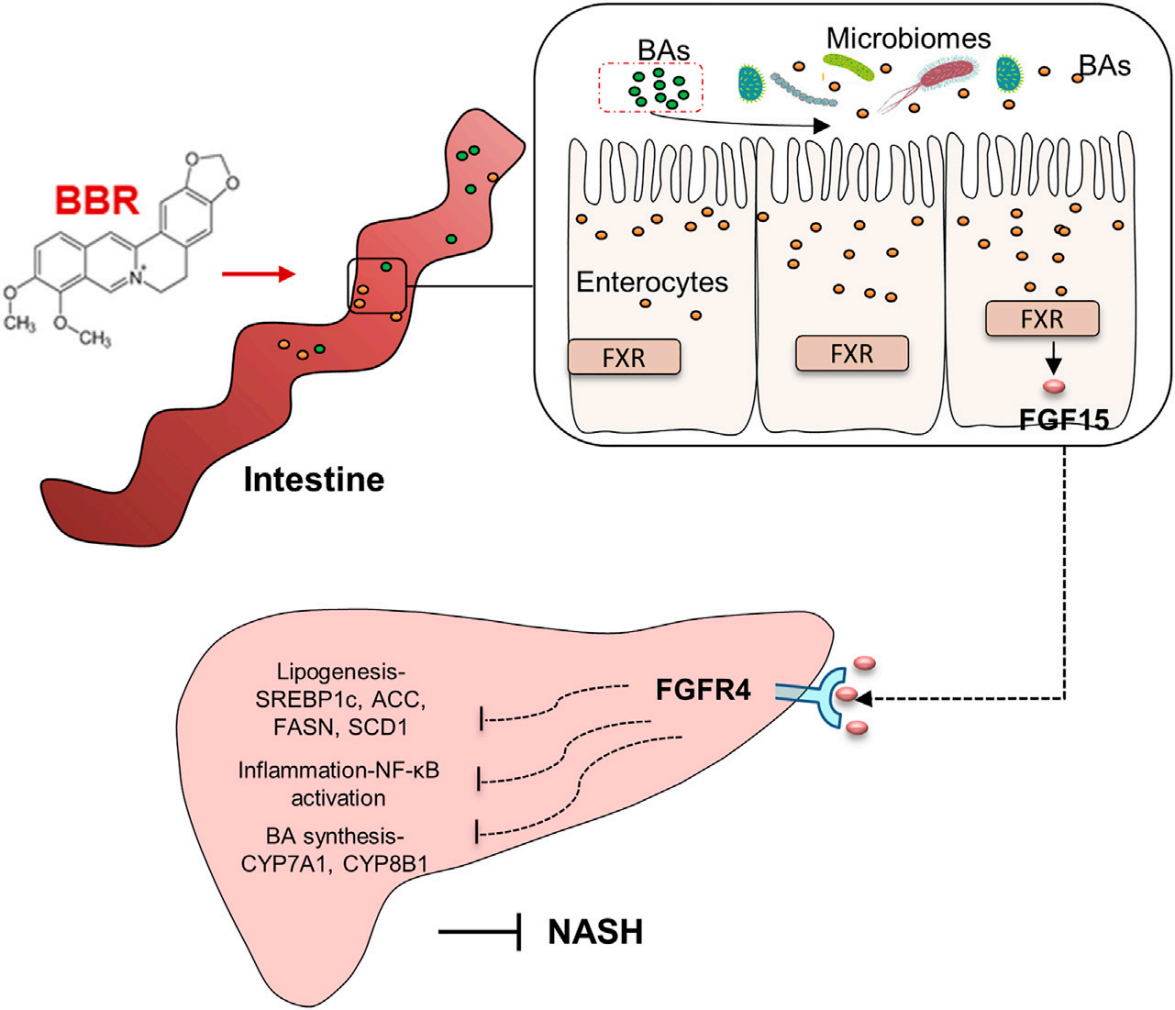
Graphic summary of the study.

The lipid-lowering and anti-inflammatory effects of BBR are of clinical value in treating NASH. In this study, we found that BBR treatment significantly improves hepatic steatosis and inflammation, as well as dyslipidemia in NASH mice, and beneficial effects are associated with the regulation of gut microbiota and BA metabolism, which further activate intestinal FXR and promote FGF15 secretion. These data reveal that intestinal FXR activation might contribute to the improvement of BBR on NASH (Figure 7).

NAFLD is considered to be the hepatic manifestation of the metabolic syndrome, and the pathogenesis of the progressive form NASH is elusive, arising a series of hypotheses, and of them, the “multi-parallel- hits” theory is the most popular one (Younossi, 2008). The theory emphasizes the pathological factors of the liver, and also from peripheral tissues (adipose, gut, etc.). Considering the characteristics of the distribution and excretion of active metabolites in the BBR pharmacokinetics study, as well as the contribution of gut-derived endotoxins to NASH development, we adopted HFD-DSS induced NASH mouse model. Studies revealed that DSS could increase portal LPS level and exaggerate the development of NASH, suggesting that the alteration of the gut environment is a potential risk for NASH (Gabele et al., 2011; Achiwa et al., 2016).

BBR is a kind of benzylisoquinoline alkaloids and reported to reshape gut microbiota in NASH patients and animals. A randomized, parallel controlled, open-label clinical trial has been conducted to evaluate the effects of BBR on NAFLD, and the 16-week BBR treatment (0.5 g, PO, *tid*) has shown better improvement in reducing body weight and serum lipid profile in comparison to pioglitazone (15 mg, PO, *qd*) in NAFLD patients (Yan et al., 2015). In mildly overweight patients with NAFLD, 1-month BBR intervention reduces body weight and improves insulin resistance (Wu et al., 2019). Under physiological conditions, administration of BBR to C57BL/6 mice is reported to decrease Firmicutes-to-Bactrodetes ratio, enrich *Bacteroides* and decrease secondary BAs (DCA, LCA, etc.) in the liver and serum of the mice (Guo et al., 2016). It is reported that Firmicutes-to-Bacteroidetes ratio increase is a typical feature of obese mice (Gu et al., 2019) and humans (Monga Kravetz et al., 2020), whereas weight loss is often accompanied by a higher abundance of Bacteroidetes. In our study, NASH mice showed increased Firmicutes-to-Bactrodetes ratio as the relative abundance of Firmicutes increased and Bacteroidetes decreased, whereas BBR treatment reversed the ratio. At the family level, BBR treatment enriched the abundance of Bacteroidetes, which is actively involved in the BSH activity. BSH catalyzes the hydrolysis of the amino group from the conjugated BAs to produce unconjugated BAs, which is a prerequisite for the subsequent secondary BA production (Enright et al., 2017). BSH-producing microbiomes such as *Bacteroides*, *Clostridium*, *Lactobacillus*, *Bifidobacterium*, *Listeria*, *Eubacterium*, *Escherichia*, *Eggerthella*, *Peptostreptococcus*, and *Ruminococcus* have been reported to be involved in BA conversion (Urdaneta and Casadesus, 2017; Jia et al., 2018). *Lactobacillus* that over-expressing BSH induces reduced cholesterol absorption and accelerated cholesterol transportation, which counters hypercholesterolemia in high-cholesterol-diet animals (Wang et al., 2019). Clinical investigation revealed that the abundance of intestinal *Bacteroides fragilis* and *Bacteroides dorei* is decreased in NASH patients compared with healthy controls (Zhang et al., 2019).

Previous studies suggest that LDL receptors, adenosine 5′-monophosphate-activated protein kinase, as well as glucokinase are involved in the actions of BBR. Due to the low bioavailability of BBR, recent studies have been concerned more about the function of BBR on the intestinal FXR. Short-term BBR exposure reduces *Clostridium* cluster XIVa and IV, and activates the intestinal FXR due to the accumulation of TCA, indicating that BBR alters BA metabolism and activates FXR signaling through directly modulating gut bacteria (Tian et al., 2019). In HFD-induced obese mice, the lipid-lowering effect, as well as the anti-obesity potential of BBR, are associated with the increase of serum TCA level and the intestinal FXR activation, however, BBR fails to prevent HFD-induced obesity in intestine-specific FXR knockout mice (Sun et al., 2017). Here we identified the altered BA species (DCA, UDCA) that in response to BBR treatment in NASH mice. Although not as efficient as CDCA in activating FXR, the DCA and UDCA species are also the confirmed FXR agonists (Li et al., 2016; Zaufel et al., 2021). In addition, TβMCA is a known antagonist of FXR in rodents (Li et al., 2013), consistently, we found that fecal TβMCA in BBR-treated mice was decreased, indicating the intestinal FXR activation might be the critical mechanism underlying the efficacy of BBR on NASH.

Animal studies demonstrated that FXR activation could reduce hepatic BA levels and protect the liver against BA toxicity (Kim et al., 2015; Li et al., 2021). FXR agonists have been proved to be effective in treating diet-induced NAFLD/NASH (Gonzalez et al., 2016; Schmidt et al., 2017; Li J. et al., 2020). In addition, FXR inhibition or knockout could promote lipid accumulation or exacerbate the NASH progression in animals. While the activation of FXR is beneficial for NAFLD/NASH patients, side effects are difficult to avoid for long-term treatment. Studies found that the selective intestinal FXR agonist fexaramine could improve obesity-related metabolic dysfunctions, and activate G protein-coupled bile acid receptor 1/Glucagon-like peptide-1 signaling to improve insulin sensitivity and increase adipose tissue browning (Jiang et al., 2015). These reports implicated that intestinal FXR activation might be a better choice for NAFLD. Actually, activation of intestinal FXR promotes FGF15 (rodents)/19 (human) production, and the secreted FGF15/19 protein can enter the liver via the portal vein. The receptors of FGFs are widely expressed in the liver, intestine, adipose, and brain. It is found that the FGF19 level in humans is directly associated with uncoupled protein1 expression in subcutaneous adipose tissue, whereas supplementation of FGF15 or FGF19 induces white adipose browning in mice (Moron-Ros et al., 2021). The FGF19 analog NGM282 is reported to reduce liver fat content in patients with NASH (Harrison et al., 2018). In the present study, we found that the intestinal FXR activation mediated FGF15 is increased in NASH mice upon BBR treatment, and the binding of FGF15 with liver-specific receptor FGFR4 may contribute to the anti-steatosis and anti-inflammatory properties of BBR. We also analyzed the FXR expression in the liver, but BBR treatment showed no obvious effect on liver FXR expression. Thus, the inhibition of BA synthesis enzymes (CYP7A1 and CYP8B1), might attribute to intestinal FXR activation.

In summary, our study highlighted the mechanisms of BBR on modulating gut microbiota and BA metabolism, and the subsequent activation of intestinal FXR in promoting FGF15 production to alleviate NASH in mice. Our study identified the activation of intestinal FXR, which is associated with gut microbiota and BA metabolism, as a potential target for BBR treatment. However, since this is a single dose study using a pretty high dose BBR in mice, the scientific interpretability is not strong enough. In addition, as the exact regulation of FXR in different metabolic disorders is still elusive, further studies are in need to clarify the exact mechanisms.

## Data Availability

The original contributions presented in the study are publicly available. This data can be found here: National Center for Biotechnology Information (NCBI) BioProject database under accession number PRJNA753953.

## Glossary

ACC: acetyl-CoA carboxylase
ALT: alanine transaminase
AST: aspartate transaminase
BAs: bile acids
BBR: berberine
BSH: bile salt hydrolase
CA: cholic acid
CDCA: chenodeoxycholic acid
CMC-Na: carboxymethyl cellulose sodium solution
CYP7A1: cytochrome P450 7A1
CYP8B1: cytochrome P450, family 8, subfamily B, polypeptide 1
DCA: deoxycholic acid
DSS: dextran sulfate sodium
FASN: fatty acid synthase
FGF15: fibroblast growth factor 15
FGFR4: fibroblast growth factor receptor
FXR: farnesoid X receptor
GAPDH: glyceraldehyde-3-phosphate dehydrogenase
H&E: hematoxylin and eosin staining
HFD: high-fat diet
IL-6: interleukin 6
LDL-c: low-density lipoprotein cholesterol
LPS: liposaccharide
MCA: muricholic acids
NAFLD: nonalcoholic fatty liver disease
NASH: nonalcoholic steatohepatitis
NF-κB: nuclear factor-κB
ORO: oil red O
OTUs: operational taxonomic units
PCoA: UniFrac distance-based principal coordinate analysis
RT-qPCR: quantitative real time-PCR
SCD1: stearoyl-CoA desaturase
SHP: small heterodimer partner
SREBP1: sterol regulatory element binding protein 1
TC: total cholesterol
TCA: taurine conjugated cholic acid
TCDCA: taurochenodeoxycholic acid
TDCA: taurodeoxycholic acid
TG: triglyceride
TNF-α: tumour necrosis factor alpha
TUDCA: tauroursodeoxycholic acid
UPLC/MS: ultra-performance liquid chromatography tandem-mass spectrometry
16SrDNA: 16S ribosomal DNA
