# Supplementary material for: Berberine Alleviates Non-alcoholic Steatohepatitis Through Modulating Gut Microbiota Mediated Intestinal FXR Activation
Source: Front Pharmacol. 2021 Sep 17;12:750826. doi: 10.3389/fphar.2021.750826 (PMC8484326; doi:10.3389/fphar.2021.750826)
Supplement: Supplementary file 1 [file DataSheet1.pdf]

Supplementary Material

Supplementary Table 1 Fecal BA levels

Metabolites (nmol/mg)	cholic acid	chenodeoxycholic acid	ursodeoxycholic acid	deoxycholic acid	tauro α-muricholic acid	tauro β-muricholic acid	tauro ω-muricholic acid	tauro-cholic acid	tauro-chenodeoxycholic acid	tauro-ursodeoxycholic acid	tauro-deoxycholic acid	tauro α-muricholic acid	tauro β-muricholic acid	tauro ω-muricholic acid
Abbreviateion	CA	CDCA	UDCA	DCA	αMCA	βMCA	ωMCA	TCA	TCDCA	TUDCA	TDCA	TαMCA	TβMCA	TωMCA
C1	0.850	2.013	0.723	0.231	8.254	35.216	8.021	3025.184	4082.659	501.901	6.892	198.138	256.039	2850.776
C2	1.056	0.956	1.058	0.562	1.025	36.014	3.019	1820.362	7833.201	1112.089	6.100	312.050	383.951	4524.918
C3	1.944	1.330	2.014	0.802	6.192	20.901	1.904	3184.240	2922.560	990.882	2.169	360.842	422.743	6712.263
C4	0.965	0.877	1.432	0.112	6.025	18.235	2.448	3882.364	8702.364	665.024	8.254	164.984	216.885	6812.281
C5	0.953	1.109	0.816	0.509	2.039	16.951	1.673	3308.276	6101.255	852.369	1.125	252.330	304.231	3968.931
C6	0.838	0.782	0.759	0.208	2.356	12.320	1.439	2306.103	3044.702	752.387	1.591	152.348	280.249	5459.919
C7	1.152	1.437	2.054	0.372	5.814	15.703	1.604	3108.627	4021.369	1109.036	6.091	288.097	339.998	3361.796
C8	0.980	1.346	0.703	0.219	1.591	15.023	1.719	2703.022	6025.870	1100.025	5.814	301.986	353.887	5071.984
M1	6.141	5.736	0.745	0.916	15.216	306.216	3.606	2350.739	862.050	711.054	5.724	385.176	801.359	2410.113
M2	3.607	4.944	2.952	1.224	14.424	614.424	4.479	3624.881	760.842	859.846	13.809	423.969	2385.237	4084.255
M3	4.807	1.013	0.979	1.507	20.493	310.493	1.650	2212.227	104.984	563.988	4.181	210.111	803.362	2271.600
M4	7.829	4.510	2.206	0.578	16.578	846.578	2.784	2560.245	852.330	651.334	5.146	285.456	2309.274	2371.618
M5	7.170	1.031	2.023	0.451	29.449	829.449	2.316	2958.894	752.348	801.352	9.447	281.474	1307.100	3128.268
M6	8.908	0.435	0.597	2.085	19.915	772.915	5.239	1109.883	1188.097	1007.101	9.069	341.223	788.625	5109.256
M7	8.116	4.235	2.144	1.016	15.115	684.415	3.117	1800.759	2001.986	700.990	8.405	555.112	784.019	2901.132
M8	6.885	3.158	1.666	1.198	14.138	594.138	3.625	3101.947	813.847	652.851	6.455	430.074	962.854	1611.320

B1	202.138	348.384	95.213	163.877	6.069	714.538	425.894	2309.890	2163.045	171.102	3.602	306.550	376.848	4521.115
B2	270.379	129.129	203.420	472.085	5.277	722.746	118.240	3984.032	2271.063	483.310	5.388	214.758	212.933	6035.257
B3	603.938	36.783	99.490	168.154	9.346	732.815	168.805	2171.378	2227.712	979.379	1.458	310.827	295.804	4350.603
B4	1020.729	386.218	65.575	274.239	6.143	1254.900	138.818	2271.396	1008.701	715.464	4.954	146.912	229.270	4482.621
B5	116.017	516.205	112.446	687.110	1.863	587.771	357.103	3028.045	2800.577	592.335	1.475	829.783	150.770	5239.270
B6	705.110	197.921	121.912	130.576	9.258	781.237	262.969	1001.034	1510.765	641.801	0.880	173.249	260.493	7220.259
B7	174.197	72.054	73.412	142.076	4.568	692.738	106.130	2800.910	2309.558	553.301	3.880	204.749	372.571	1012.135
B8	422.258	498.893	83.134	151.799	3.491	662.460	181.825	1511.098	3903.700	463.024	6.181	594.471	680.779	3722.323

C-CON group, M-NASH group, B-BBR group

Supplementary Table 2 Serum BA levels

Metabolites μM)	cholic acid	chenodeoxycholic acid	ursodeoxycholic acid	deoxycholic acid	tauro α-muricholic acid	tauro β-muricholic acid	tauro ω-muricholic acid	tauro-cholic acid	tauro-chenodeoxycholic acid	tauro-ursodeoxycholic acid	tauro-deoxycholic acid	tauro α-muricholic acid	tauro β-muricholic acid	tauro ω-muricholic acid
Abbreviateion	CA	CDCA	UDCA	DCA	αMCA	βMCA	ωMCA	TCA	TCDCA	TUDCA	TDCA	TαMCA	TβMCA	TωMCA
C1	0.084962	0.009158	0.008935	0.431000	0.061200	0.197492	0.023406	0.096374	0.185672	0.435428	0.466385	0.082586	0.014753	0.021685
C2	0.655534	0.085689	0.085466	0.561900	0.060678	0.150607	0.014757	0.065860	0.238759	0.964800	0.622932	0.064371	0.017295	0.031131
C3	0.104379	0.090667	0.010444	0.802300	0.069097	0.207452	0.018977	0.056640	0.163162	0.859645	0.766858	0.297829	0.021292	0.039815
C4	0.045490	0.005978	0.005756	0.501300	0.066877	0.162289	0.012968	0.063135	0.140320	0.576945	0.294213	0.145071	0.008169	0.011296
C5	0.095270	0.131663	0.003144	0.342100	0.073343	0.127184	0.011153	0.067682	0.156413	0.739478	0.229325	0.171114	0.006367	0.007381
C6	0.048385	0.007147	0.045242	0.168300	0.061836	0.057756	0.012432	0.074946	0.167678	0.652738	1.061021	0.130914	0.029459	0.057564
C7	0.105230	0.096362	0.034140	0.321900	0.077142	0.120660	0.013327	0.315787	0.782337	0.962151	0.516820	0.174857	0.014349	0.024728
C8	0.060067	0.066693	0.066471	0.200100	0.062138	0.047712	0.062181	0.118869	0.294489	0.954334	0.609598	0.215257	0.016925	0.030326

M1	0.144307	0.016845	0.149594	0.084234	0.521572	0.332069	0.227173	0.133519	0.345000	0.966578	2.049411	0.169735	0.046422	0.060381
M2	0.116998	0.163972	0.271754	0.033573	0.257363	0.171147	0.135178	0.133087	0.304496	1.168841	0.506306	0.061793	0.027954	0.102324
M3	0.199954	0.122249	0.034214	0.042401	0.196648	0.343407	0.157190	0.133191	0.042016	0.766663	1.227298	0.427847	0.031656	0.056911
M4	0.106876	0.222079	0.047995	0.033797	0.341921	0.173537	0.156375	0.133187	0.341110	0.885397	0.587518	0.298771	0.036256	0.059417
M5	0.076646	0.191401	0.213649	0.032037	0.297279	0.473160	0.194228	0.133365	0.301096	1.089326	0.785859	0.110740	0.077459	0.078374
M6	0.282166	0.039222	0.048829	0.045517	0.526530	0.463587	0.071544	0.132788	0.475487	1.369013	1.755354	0.031545	0.037884	0.128004
M7	0.090984	0.174595	0.020613	0.050821	0.106062	0.431989	0.120729	0.133019	0.801213	0.952897	2.966760	0.331218	0.024970	0.072683
M8	0.383365	0.039903	0.160649	0.065410	0.104986	0.382526	0.100447	0.132924	0.325709	0.887459	0.337206	0.442694	0.042818	0.040369
B1	0.000000	0.473903	0.289430	0.031005	0.036709	0.111354	0.189739	0.915493	0.568658	0.611447	0.682230	0.252336	0.038061	0.009169
B2	0.931272	0.001219	0.000141	0.024974	0.014516	0.519549	0.071422	0.344612	0.564038	0.721213	0.839857	0.732636	0.069547	0.033721
B3	0.000000	0.000852	0.000161	0.024597	0.060084	0.195570	0.057906	0.217275	0.257350	0.551778	0.322220	0.377797	0.038498	0.040958
B4	0.000000	0.000316	0.000344	0.024387	0.045820	0.123305	0.039571	0.279397	0.570224	0.736988	0.251154	0.264671	0.021753	0.054675
B5	0.000000	0.000090	0.000168	0.027771	0.063114	0.158560	0.034032	0.190933	0.508074	0.907266	1.162021	0.383068	0.030219	0.039413
B6	0.000000	0.000484	0.000206	0.024853	0.017572	0.103873	0.045031	0.196217	0.385786	1.255288	0.510781	0.213515	0.026612	0.065410
B7	0.000000	0.000944	0.000111	0.026879	0.049374	0.108356	0.037935	0.164203	0.340991	0.348082	0.566017	0.181056	0.023422	0.040609
B8	0.000000	0.001262	0.000190	0.029478	0.038694	0.093186	0.040667	0.183035	0.437052	0.271313	0.667627	1.022638	0.015402	0.047464

C-CON group, M-NASH group, B-BBR group

Supplementary Table 3 Liver BA levels

Metabolites (ng/mg)	cholic acid	chenodeoxycholic acid	ursodeoxycholic acid	deoxycholic acid	tauro α-muricholic acid	tauro β-muricholic acid	tauro ω-muricholic acid	tauro-cholic acid	tauro-chenodeoxycholic acid	tauro-ursodeoxycholic acid	tauro-deoxycholic acid	tauro α-muricholic acid	tauro β-muricholic acid	tauro ω-muricholic acid
Abbreviateion	CA	CDCA	UDCA	DCA	αMCA	βMCA	ωMCA	TCA	TCDCA	TUDCA	TDCA	TαMCA	TβMCA	TωMCA
C1	0.018419	0.009655	0.016924	0.005765	0.057957	0.051861	0.039769	8.090143	1.659384	0.420422	0.001747	0.018419	0.872347	18.354465

C2	0.014357	0.017151	0.016582	0.004796	0.066982	0.045778	0.048928	14.850217	1.636236	0.237968	0.002305	0.014357	0.798909	4.194853
C3	0.066424	0.011599	0.015451	0.010848	0.025125	0.072292	0.061491	14.256472	1.983565	0.143575	0.003764	0.066424	0.992525	1.770816
C4	0.032355	0.008803	0.013682	0.006455	0.040765	0.121814	0.042801	13.211900	3.226920	0.142737	0.002138	0.032355	0.904235	17.237683
C5	0.038163	0.008234	0.011877	0.007506	0.070220	0.049520	0.039861	10.147577	2.500064	0.175295	0.001187	0.038163	0.767099	12.851544
C6	0.029197	0.008862	0.006122	0.007467	0.053215	0.052453	0.043415	8.998759	2.239067	0.145894	0.001776	0.029197	0.684224	23.346205
C7	0.038998	0.008401	0.012283	0.009275	0.082058	0.046295	0.052500	21.708226	2.464570	0.434251	0.003009	0.038998	0.401861	20.121211
C8	0.048008	0.011935	0.006207	0.003416	0.050079	0.006388	0.034436	6.571905	1.221394	0.146258	0.001674	0.048008	0.535600	4.123222
M1	0.087420	0.009544	0.015823	0.002781	0.149962	10.603037	0.068316	23.498781	0.775175	0.349970	0.326779	1.381419	0.599295	3.188400
M2	0.083599	0.016351	0.009322	0.002604	0.125494	16.890963	0.198350	42.938023	2.850856	0.848336	2.262561	1.564367	0.640305	2.436213
M3	0.090171	0.038298	0.009828	0.003894	0.046374	9.844135	0.102283	23.768526	3.462635	0.406106	1.579979	1.791654	0.691254	4.235961
M4	0.091207	0.010633	0.023906	0.001850	0.130992	14.725483	0.071655	13.430088	4.622288	0.543204	0.585623	3.827836	1.991312	3.682901
M5	0.092478	0.015180	0.034134	0.002572	0.265442	3.983347	0.103710	18.656939	3.332043	1.213341	0.166816	1.872132	0.709294	6.523022
M6	0.158362	0.012483	0.004769	0.001696	0.193913	13.539170	0.049018	14.460500	3.433153	2.050693	1.751563	1.233939	1.156774	4.287261
M7	0.074174	0.032112	0.021660	0.002146	0.160541	4.031444	0.276863	9.509374	4.012656	0.233085	2.341076	2.115934	2.898316	1.300640
M8	0.098588	0.023647	0.008862	0.001513	0.173948	2.403781	0.057806	16.429769	5.529858	1.416600	0.897602	2.294055	0.550785	6.461602
B1	0.272546	0.043309	0.018081	0.001861	0.237974	2.351145	0.367851	15.076352	1.624947	0.116448	4.103895	0.727841	10.101545	1.283266
B2	0.338598	0.020575	0.026451	0.004528	0.029561	2.404422	0.138467	9.071980	3.117708	0.258020	3.632074	1.146283	15.148044	2.036875
B3	0.623725	0.028601	0.050333	0.006465	0.178532	1.395440	0.087302	15.869023	1.163214	0.229898	1.291551	1.325517	16.678520	3.021500
B4	0.309714	0.018864	0.035794	0.000903	0.173717	1.217449	0.112263	19.348207	3.463645	0.154295	4.914694	0.606053	8.556795	3.066523
B5	0.305611	0.023864	0.020388	0.004102	0.058790	1.131712	0.076718	16.487175	2.428372	0.197761	0.670039	0.926908	12.002840	1.786599
B6	0.268977	0.016827	0.018978	0.001678	0.067935	0.822526	0.065978	11.492728	1.211828	0.174564	0.947392	0.559634	11.056676	2.457762
B7	0.369639	0.030922	0.051338	0.002997	0.167617	1.048406	0.073544	15.492200	1.600553	0.257312	3.627013	1.058295	13.413971	1.513300
B8	0.314390	0.028960	0.017563	0.001765	0.045874	1.002980	0.078841	13.470820	2.398368	0.255221	3.461662	1.109314	13.961923	2.283134

C-CON group, M-NASH group, B-BBR group

Supplementary Figure 1

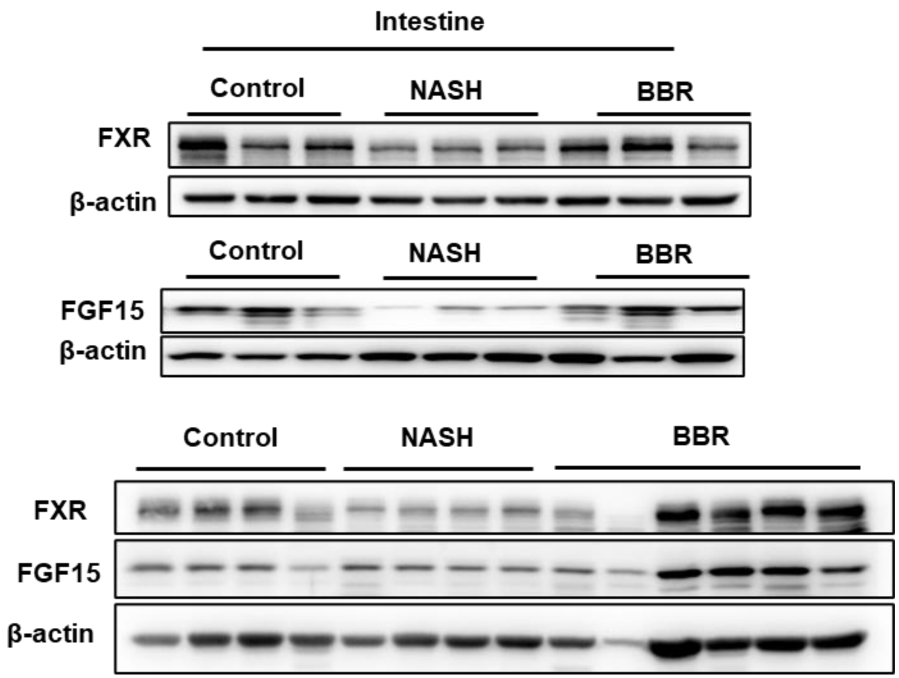

Figure S1. Repeats of Figure 4A

Supplementary Figure 2

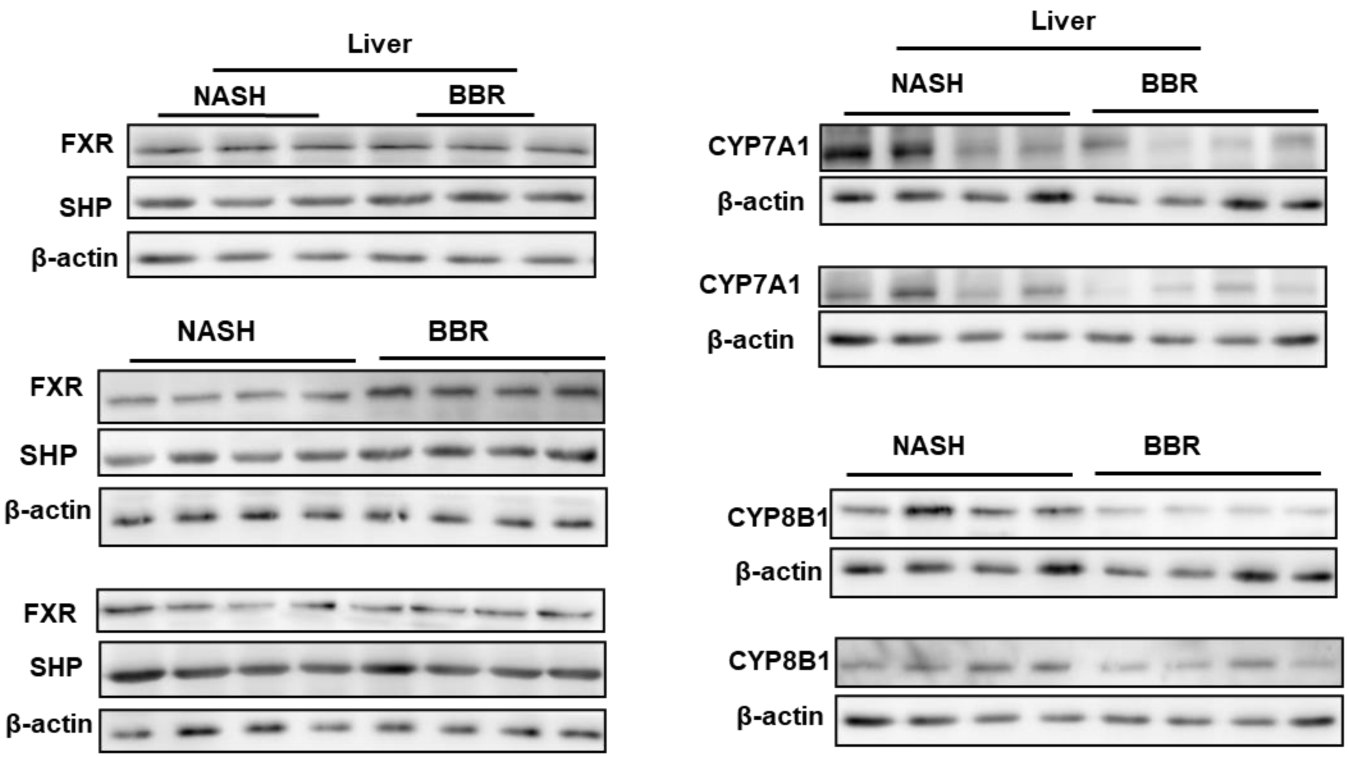

Figure S2. Repeats of Figure 4D

Supplementary Figure 3

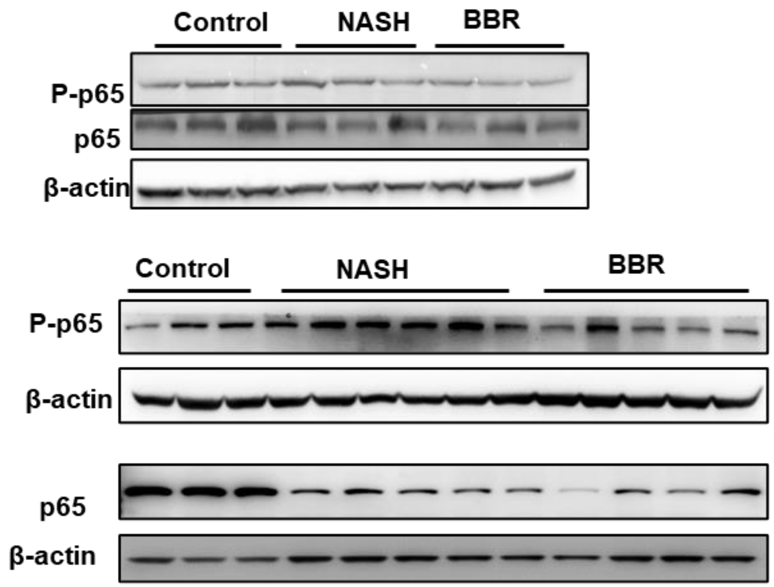

Figure S3. Repeats of Figure 5B
